# Existential Transformational Game Design: Harnessing the “Psychomagic” of Symbolic Enactment

**DOI:** 10.3389/fpsyg.2020.571522

**Published:** 2020-11-04

**Authors:** Doris C. Rusch, Andrew M. Phelps

**Affiliations:** ^1^Games and Society Lab, Department of Game Design, Uppsala University, Campus Gotland, Uppsala, Sweden; ^2^Human Interface Technology Laboratory, College of Engineering, University of Canterbury, Christchurch, New Zealand; ^3^American University Game Lab, Division of Film and Media Arts, School of Communication, American University, Washington, DC, United States

**Keywords:** game design, transformation, existential, symbolic action, metaphor, meaning, psychology, experiential

## Abstract

This paper explores indexical symbolic enactment as a way to promote authenticity and inner balance through digital games. Drawing on research from cognitive anthropology, neurobiology, and psychology, the following presents an argument for *why* and *how* indexical symbolic enactment can impact us on a deep, unconscious level and contribute to personal transformation. It identifies four high-level guidelines that can inform existential transformative game design: (1) ritual theming (i.e., liberation, transformation, and commemoration/celebration); (2) metaphorical approach; (3) contextual mechanisms that promote a readiness for change and processing; and (4) psychological resonance. It then uses these guidelines as an analytical lens for a case study on the game *Papo & Yo* (Minority Media Inc., 2012). This analysis shows how indexical symbolic enactment can contribute to a game’s transformative potential and examines missed opportunities when design decisions emphasize gameplay considerations rather than symbolic congruency and psychological resonance.

## Introduction

How can we intentionally design for deep (in the sense discussed by Rusch, 2016), transformative gameplay experiences that contribute to a meaningful life for players? Adopting a perspective informed by existential psychotherapy, a meaningful life is one in which one has faced and accepted the inevitability of death, developed a sense for one’s purpose or calling, focused on making self-directed choices that are in alignment with one’s true nature, and cultivated quality connections to something beyond oneself (Existential Psychotherapy, Yalom, 1980). Further, existential psychotherapist Bugental (1990, p. 246) writes: “Viewed from an existential perspective, the good life is an authentic life, a life in which we are as fully in harmony as we can be. Inauthenticity is illness, is our living in distorted relation to our true being.”

Recently, Rusch (2020) discussed in detail the various goals of existential psychotherapy and how they can inform game experience goals, while Phelps has focused on encoding “deep” meanings through experiential and proceduralized gameplay (Phelps et al., 2020). This article extends these ideas and focuses on the idea of a meaningful life from the perspective of authenticity and inner balance, and specifically on “symbolic enactment.” Symbolic enactment is an intrinsic component of pretend play and can take very banal forms, such as a banana symbolically representing a telephone. This is an iconic representation; the banana simply functions as a placeholder for another, well-understood thing. We have spoken about plenty of bananas and have rarely been transformed by it. Thus, iconic symbolic representation alone is insufficient; symbolic enactment is based instead on *indexical* symbols: objects and actions that do not refer merely to something known and easily graspable, but to intangible concepts and the psychologically ineffable. This is where the design focus is “deep,” because what is enacted refers to something below the surface of physical, conscious reality. By surfacing it through symbols – actions and objects that are acted upon – we can connect with the ineffable and affect personal changes on a profound and otherwise elusive level.

Symbolic enactment in the broader sense is an inherent characteristic of ritual, theater, and games. Yet it is mostly ritual and theater that have embraced and leveraged indexical symbolism for personal transformation and growth. There is significant research on the use of ritual in psychotherapy (Parker and Horton, 1996; Kirmayer, 1999; Goodwyn, 2016). Alejandro Jodorowski’s “psychomagic” poetic acts have become famous as a form of theater counseling that is based on symbolic action (Jodorowski, 2010, 2015). In digital games, however, symbolic enactment has remained largely underexplored and under-used. The Games for Change discourse has mainly focused on a kind of learning through games that favor rationality. Embodied experience (Gee, 2003) is lauded as a potent aspect of change affected through games, but mostly as a vehicle of sense-making. It emphasizes the *understanding* of whatever is represented through the ruleset (Gee, 2003). Procedural rhetoric also champions cognitive understanding by focusing on the messages and ideas expressed through rules and mechanics (Bogost, 2007). Favoring the conscious aspect of learning and change – as would be expected in our modern, rational, Western world – has limited explorations of how to address the unconscious, and the subtler, more elusive kinds of inner shifts that can be ignited by engaging in various artistic and expressive experiences such as videogames. Humans are far from being purely rational creatures. If transformative game design approaches do not take that into account, they are missing out on much of games’ potential to have a real and lasting impact. However, when we stray from the currently predominant model of transformative game design (Culyba, 2018), which focuses on specific changes that are imposed on players through the game and are supposed to be clearly predictable and measurable, there are very few guidelines. We leave the well-lit path of reason and quantifiable results at our own risk.

Please note that this work is neither claiming successful treatment of mental illness through games nor recipes for the design of games as therapeutic tools. Our intention regarding an existential, transformative game design framework is to suspend the requirement for hard proof of whether a game “works” – at least for now – for the sake of exploring new opportunities for games to tap into thus far underexplored transformative potentials. Our method is to draw from a broad range of research sources, including cognitive anthropology, existential and depth psychology, neurobiology, and game design/studies, to craft an argument for how and why symbolic enactment can be psychologically powerful and how it can inform transformative gameplay experiences. Further, we identify several guidelines inspired by this research that game designers can use to design transformative games with a special focus on symbolic enactment. These guidelines then act as an analytical lens for the case study of *Papo & Yo* (Minority Media Inc., 2012) to discuss opportunities and challenges of symbolic enactment in the context of videogames. While a bit dated, *Papo & Yo* lends itself to this analysis, because (a) the game deals with an inner conflict – the emotional struggle of a boy to “let go” of the desire to save his alcoholic father, (b) it uses ritualistic/spiritual motifs in its narrative and mechanics, and (c) it was intentionally created to promote transformation and healing.

## Authenticity and the Unconscious

To design for authenticity, personal integrity, and inner balance, it is useful to explore what helps and hinders these feelings. Game design – as a means to evoke a specific experience, communicate a message, or promote some kind of change – is often understood as problem solving (Culyba, 2018). Yet, a basic principle of (particularly but not only depth) psychology is that the experience of an authentic life (as described by Bugental, 1990 above) depends on the alignment of *all* parts of ourselves, both the feeling and thinking self, or – as it is more commonly referred to – the conscious and unconscious mind.

In his book *Inner Work*, Johnson (1986) writes:

The unconscious is an enormous field of energy, much larger than the conscious mind. Jung compared the ego—the conscious mind—to a cork bobbing in the enormous ocean of the unconscious (…). Deep in this unseen ocean of energy huge forces are at work. Mythical kingdoms, symbolized by the legends of Atlantis, exist there in the depths and carry on lives parallel to the daily life of our conscious minds. Centers of alternative consciousness, alternative values, attitudes, and ideas exist there like other islands in the great sea. (…) The purpose of learning to work with the unconscious is not just to resolve our conflicts or deal with our neuroses. We find there a deep source of renewal, growth, strength, and wisdom. We connect with the source of our evolving character; we cooperate with the process whereby we bring the total self together. (pp. 8-9)

Getting in touch with the unconscious and becoming accepting of its contents is a path toward a more authentic and integrated person. Apart from various psychotherapeutic methods such as active imagination (Jung, 1997), dreamwork (Jung, 2002) and sand play therapy (also invented by Jung but further developed by Kalff, 2003), an essential means of communication between, and alignment of, the conscious and the unconscious mind since the dawn of humankind, have been myth. Existential psychotherapist May (1991, p. 15) noted: “A myth is a way of making sense in a senseless world.”. This corresponds to the view of Campbell (2004, p. 3) on myth as a vehicle to “reconcile consciousness to the preconditions of its own existence; that is to say, the nature of life.” Myth, *via* imagery and symbolism, speaks the language of the unconscious, acts as a map through our own unknown forests, and provides personal navigation and calibration tools (Campbell, 2004).

An example of using this kind of myth-based approach to imagery and symbolism in media is *Women Who Run with the Wolves* book of Estés (1996), in which the Jungian psychologist analyzes a range of myths based on the “Wild Woman” archetype. Her detailed dissection of stories such as *Bluebeard’s Brides*, *The Seal Skin*, and *The Red Shoes* makes their symbolism apparent and explains how they exemplify common psychological patterns. *Bluebeard’s Brides* appeals to honoring our instincts – if something feels “off,” it probably is. *The Seal Skin* speaks to the need to “go home to ourselves.” If we leave our skin behind and live a life in an environment that does not fulfill our needs, like a sea creature forced to live on land, we dry out and die. *The Red Shoes* reminds us of the value of our own creativity, of investing psychic energy into something that fulfills us rather than filling our lives with substitute pleasures that leave us constantly hungry (forever driven/dancing) and never satisfied. Each of these myths thus, from a story perspective, seeks to provide the listener with a deep sense of authenticity not purely conveyed through the story itself but also through the alignment of the narrative with subconscious elements of the human condition.

The power of myth is that it works without having to analyze it intellectually. If the recipient is in the right mindset – open to the themes the myth deals with – the symbolism and imagery within the story “resonate” and activate the recipient’s imagination. This sets a transformative process in motion that is uncoerced and out of the recipient’s own accord (Campbell, 1991). As Jung notes: “The auditor experiences some of the sensations but is not transformed. Their imaginations are stimulated: they go home and through personal fantasies begin the process of transformation for themselves” (Bonnett, 2006, p.27).

Myth, dreamwork, and active imagination – all of these are instruments to surface what slumbers in the depths of our unconscious: to raise awareness for potential needs, wants, desires, inner conflicts, and for and potential action. For example, myth is used in strategic and narrative psychotherapies to

(1) Join[…] with patients by accepting their initial framing of the problem; (2) work[…] within the patients’ own metaphors, stories and worldview to reframe, reattribute or otherwise transform the problem; and (3) [assume] that manipulation of symbols through imaginal dialog and ritual enactments can reorganize cognitive schemas, unconscious dynamics and interpersonal interaction (Kirmayer, 1999, p. 451).

As noted by Rusch (2018, p. xix), myth provides relevant context to symbolic action in the sense that “[r]itual is simply myth enacted; by participating in a rite, you are participating directly in the myth,” which is based in turn on Campbell (2004). This provides a background for recognizing the criticality of symbolic enactment as a huge change agent. This “acting” does not only refer to behavior change once an issue is conscious. At first, it refers to an action that further strengthens the dialogue with the unconscious by addressing it directly, acknowledging its messages and sending messages back to it; s*ymbolic* action speaks the language of the unconscious. Johnson proposes performing a ritual to honor the insights that appear through dreamwork and active imagination (Johnson, 1986). The unconscious must know that we are paying attention, and actions speak louder than words. Symbolic action has been effectively used in ritual and ritual-like performances such as poetic acts of Jodorowski (2010) in the context of psychotherapy (e.g., psychodrama) and counseling (Gilligan, 1993; Bewley, 1995; Al-Krenawi, 1999; Kirmayer, 1999; Cole, 2003). Games also have an inherent potential for symbolic enactment but there is almost no systematic inquiry of such.

## Iconic and Indexical Symbolic Action

In exploring symbolic action in detail, the nuances of various interpretations of these concepts are critical. The following is a discussion of the terms iconic, indexical, symbol, and sign and how they are used by Peirce as well as Jung. Although Peirce’s work on semiotics provides the (commonly understood) basis for these terms, Jung gives them a new spin, one that is particularly relevant for the design methodology, we propose but that differs slightly from more generalized definitions as put forward by Peirce.

There are different kinds of symbols/symbolic actions. One commonly found in pretend play is *iconic* symbolic action, wherein an icon is a sign that shares some quality or likeness with that which it represents: “icons serve to convey ideas of the things they represent simply by imitating them” (Peirce, 1998, p. 5). The stick is not the sword, the broom is not the horse, the banana is not the phone, but sword, horse, and phone are all well-known entities and stick, broom, and banana share enough likeness with them to act as placeholders during play. This kind of pretend play, however, is not deep in the sense established by Rusch (2016). Rusch notes that by modeling complex abstract concepts by way of metaphors, they have a deeper meaning beyond what they represent on the surface, thus allowing the exploration of intangible aspects of the human experience such as inner processes. The concepts “phone conversation,” “horse riding,” or “sword fighting” in pretend play are anchored, and usually exhaust themselves, in the physical realm and thus carry little symbolic weight. Jung does not even dignify these types of symbols with the term “symbol” – he refers to them simply as “signs” (Sharp, 1991, p. 131). He reserves the term “symbol” for representations of “metaphysical concepts” – something deeper, more elusive and essentially unknown, e.g., inner processes. He states that

The interpretation of the cross as a symbol of divine love is *semiotic*, because “divine love” describes the fact to be expressed better and more aptly than a cross, which can have many other meanings. On the other hand, an interpretation of the cross is *symbolic* when it puts the cross beyond all conceivable explanations, regarding it as expressing an as yet unknown and incomprehensible fact of a mystical or transcendent, i.e. psychological, nature, which simply finds itself most appropriately represented in the cross. (Sharp, 1991, p. 131)

To further clarify the distinction between “sign” and “symbol” in the Jungian sense, it helps to draw on Peirce’s term “index.” An index, according to Peirce (1998), is a sign that refers to something else by being physically connected with it, for example, sweat that indicates body heat or foot prints that indicate someone has walked this path. As such, indices themselves are not terribly deep either. But when they are not reduced to referring to something physical, and instead applied to the metaphysical, the elusive, the intangible, and the ineffable, it gets much more interesting and leads Jung to elevate a sign to a symbol. Thus, it is these kinds of *indexical symbols* that are of interest when we explore the transformational potential of symbolic enactment. Indexical symbolic action can therefore be understood as a process of projecting salient aspects of an internal landscape outward, manifesting them through physical gestures and objects that represent something otherwise abstract. The tangible, symbolic manifestation of elusive ideas now allows their manipulation in a manner that the unconscious accepts authentically as “real.” “As if” becomes “as.” This can have powerfully transformative effects on the performer’s inner world.

### The Transformative Power of Indexical Symbolic Enactment

But how can indexical symbolic enactment contribute to personal transformation directly? Goodwyn (2016), referring to Kradin, explains that psychosomatic symptoms are a failure of symbol formation:

(…) patients who remain unable to psychologize (i.e. transform into a narrative of personal meaning) their symptoms stay resistant to treatment. In this context, by “symbols” we mean concrete images/objects that are iconic and indexical signs of more abstract, difficult to verbalize, and frequently highly emotional ideas. Manipulating them in a ritual manner is a way of accessing deep emotional issues in a very physical, embodied way that cannot be duplicated by mere verbal discourse. (p. 53)

While the above also includes iconic signs, the ineffable quality of indexical symbols is still our critical concern. In psychotherapy, symbol-formation makes (otherwise intangible) psychic energy available for meaningful work and personal change. The concrete, physical enactment of elusive, psychological concepts is at the heart of experiential psychotherapies (in contrast to talk therapies): such as psychodrama (Moreno, 2008), play therapy (Kalff, 2003), and ritual and ritual-like performances such as poetic acts of Jodorowski (2010). Anthropologists Thompson et al. (2009, p. 134) emphasize the importance of performance for any kind of transformative, symbolic work: “We suggest that the efficacy of mental practice resides in its performativity – that is, *doing* (even in the mind’s eye) *makes it so*.”

#### “To Die and Be Reborn”: An Example of a Poetic Act by Alejandro Jodorowski

As an example, consider one of Alejandro Jodorowski’s poetic acts, designed to help a person “who cannot free themselves from the feeling that they have failed in their professions, in love, the family, in their projects” (Jodorowski, 2015, p. 44) and, if it were not for their self-acclaimed “cowardice,” would commit suicide (Jodorowski, 2015, p. 44). Jodorowski (2015, p. 44) states that a client in such a state cannot be cured and offers the shocking conclusion: “[t]he only thing left to do is die to be reborn a new person.” He goes on to describe an elaborate process of symbolic death and rebirth, involving hiring a couple of collaborators who will dig a shallow pit, the consultant reading their own funeral speech, undressing, wrapping themselves in a sheet, lying in the pit and being covered with dirt by the collaborators, surrounded by 10 burning candles. When ready, the consultant says “I am ready to be reborn!” upon which the collaborators will dig them out and wash them with blessed water and give them clean, white clothes. This is followed by the consultant choosing a new name (on new business cards) and writing their old name on a piece of parchment and burying it with their old clothes in the pit, and erasing the key burdens and traces of the old life to make room for a new identity to settle in.

This poetic act is not mere pretend play. Its various symbolic actions are indexical rather than iconic; the feigned death is not about pretending to be physically dead, but it refers to the more elusive concept of letting go of an old self. The use of new names on business cards in the ritual fits with the power of names drawn from fairy tales; they can render threats harmless as in *Rumpelstilskin*! (Grimm and Grimm, 2013) or imbue a princess with life as in the *Never Ending Story* of Ende (1983). Every single step in this “identity rebooting” process has a deeper meaning that is made concrete and accessible through symbolic action.

The description of this act stems from *Manual of Psychomagic* of Jodorowski (2015), which is a collection of poetic acts he actually prescribed to his clients and which has been reported as successful by the clients themselves. What “successful” means is, of course, worthy of further inquiry, particularly if attempting to prove clinical relevance. But for the purpose of exploring these acts in theorizing models for deeper game design, this theory needs to address *why* such a bizarre performance *could possibly work at all*. You cannot *really* die and be reborn. As Thompson et al. (2009, p. 134) said, however: “doing (even in the mind’s eye) makes it so.”

#### Magical Consciousness

According to Greenwood and Goodwyn (2016), there are two kinds of consciousnesses: rational consciousness, which has become dominant in the western world, and *magical* consciousness. While the rational mind focuses on logical, causal relationships between actions and consequences, magical consciousness emphasizes analogical, associative thinking that is based on perceived, meaningful similarities between things. Our unconscious cares much less about logical relationships than about magical/associative/analogical ones and we have little control over that. Both kinds of thinking are important tools for meaning-making and much can be gained from using them in complement. Magical consciousness has received little attention in research overall, yet it is essential to understand the transformative power of symbolic action. It also plays a key role in the placebo phenomenon. While the kind of indexical symbolic enactment as exemplified through one of Jodorowski’s poetic acts may sound like hocus-pocus, it is not that much different from how medical treatment began.

Originally, medicine was not based on sound knowledge of the human body or what substances could really provide cures. Instead, it began with magical thinking that led to the most absurd (from a clinical point of view) concoctions and procedures to promote health. The substance *bezoar*, for example, was believed to contain the crystallized tear from the eye of a doe bitten by a snake (Benedetti, 2009, p. 4). It was the symbolic meaning healers of the time ascribed to these substances that led to their use in treatment and belief in these meanings by patients that made such substances effective. Therefore, “[t]he history of medical treatment is basically the history of the placebo effect” (Shapiro and Shapiro, 1999, p. 13), but this does not mean such effects are the equivalent of “no therapy” (Koshi and Short, 2007, p. 13). Rather, placebo describes the psychobiological phenomenon where the body and brain anticipate and participate in clinical improvement, and placebo *responses* have been observed in a variety of mental and physical disorders (see a thoroughly referenced account of these studies in Goodwyn, 2016, p. 28).

Goodwyn (2016) explains that the key to the “magic” of its effectiveness lies in the mind-body connection. Referring to biogenetic structuralist Charles Laughlin, Goodwyn writes:

Biogenetic structuralists (…) argue that the brain plays a fundamental part in ritual experience through its capacity to *co-create* a *cognized and highly symbolic* world—that is, one in which the mind participates through its various biological mechanisms. (p.26)

Laughlin himself explains: “The symbolic function amounts to the relationship between a sensory object and neurocognitive, neuroendocrinal, neuroimmunological, or other somatic processes intent upon this object” (quoted in Goodwyn, 2016, p.26). This means that powerful, complex physiological mechanisms are set in motion when we ascribe a special, symbolical meaning to a tangible object or process. Digging a shallow grave, laying in it and emerging from it “reborn” is thus felt on a biological level, causing inner shifts that in turn impact the mind. Goodwyn, still referencing Laughlin, goes on to explain that symbolically rich rituals (which are most of them),

intensify right-brain processing to produce an ‘alternative reality’ rich in intuitive, vivid sensory and emotional experience (1990,30-31)—types of experience that we are constantly trying to access in psychotherapy, and that are particularly difficult to access due to the typically low-ritual, abstract, and verbal nature of most psychotherapy modes. (p. 26)

Thus, symbolic acts are particularly conducive to envisioning – through the tangibility of bodily experience – new ways of being by utilizing the powerful interaction between body and mind. This idea is well represented in psychological literature and has played a key role in many aspects of psychotherapy (Benedetti, 2009, p.16). Key to the psycho-biological conceptualization of placebo is the role of symbolic meaning. From this perspective, the placebo effect can be defined as

a change in the body, or the body-mind unit, that occurs as a result of the symbolic significance which one attributes to an event or object in the healing environment. This definition is embedded in the notion that symbols induce expectations of an outcome, thus highlighting the crucial role of meaning and expectation. (Benedetti, 2009, p. 16)

That mental images alone can induce physiological changes is particularly noteworthy in regards to the transformative power of symbolic enactment in games. You do not have to literally touch or manipulate something to reap the benefits of symbolic action. The virtual, embodied experience within a videogame, accompanied by a vivid, sensual, emotionally evocative audio-visual design can – according to the presented research from placebo studies – be highly effective.

### Magical Realism and Symbolic Enactment

It is critical to note the role of magical realism in all of the above. Magical realism is, essentially, a subgenre of fantasy that involves the use of magic as an element in an otherwise normally functioning world. The origins of the term and the movement it describes are defined as

a term used in 1925 by a German Art Critic, Frans Roch, to indicate the demise of Expressionism, magical realism grew to become an important feature of the Boom literature of the 1960s in Latin American (particularly in Gabriel Garcia Marquez’s One Hundred Years of Solitude of 1967) until it became, in the 1990s, in the words of Homy Bhabha ‘the literary language of the emergent postcolonial world. (Hart and Ouyang, 2005, p. 1)

For our purposes regarding game narratives, actions, and symbolic enactments, it is useful to examine this in the context of Faris’ framework, who notes:

As a basis for investigating the nature and cultural work of magical realism, I suggest five primary characteristics of this mode. First, the text contains an ‘incredible element’ of magic; second, the descriptions in magical realism detail a strong presence of the phenomenal world; third, the reader may experience some unsettling doubts in the effort to reconcile two contradictory understandings of events; fourth, the narrative merges different realms; and, finally, magical realism disturbs received ideas about time, space, and identity. (Faris, 2004, p. 7)

In this context, the core use of magical realism as a construct is to set up a scenario that “involves the intentionality implicit in the conventions of the two modes” (Zamora and Faris, 1995, p. 3). Magical realism takes the conventions of the normally functioning world and a consistent but magical system and then uses the juxtaposition between these modes as an exploration of the self. This is described by Hart and Ouyang (2005, p. 2) as a “tension between surface and innerness” and has been used in numerous cultural contexts to promote and explore the concept of the self. These include Japanese notions of individuality in the fiction of Murakami Haruki (Strecher, 1999, p.263), American notions of the individual in consideration of debt and poverty *via* the game *Kentucky Route Zero* (Martens, 2020), and numerous expositions of the self and the world through games created for the Triennale Game Collection in Milan (Ingalls, 2016). Magical realism is perhaps best known in game design circles *via* the game Life is Strange (Dontnod Entertainment, 2015), which reached a wide commercial audience, and as noted by Turpin, makes clear that the “challenges for developers attempting to turn magical realism into a gaming experience are significant” (Turpin, 2017, para. 20). These are just a few examples of magical realism as applied to recent games and popular media, which illustrate a unique characteristic of this movement, namely “its ability to express a world fissured, distorted, and made incredible by cultural displacement” (Hart and Ouyang, 2005, p.6) that has allowed magical realism to “migrate around the world” (p. 11).

But, as previously noted, nowhere was this movement more vibrant than in Latin America as magical realism also has strong ties to postcolonialism. Magical realism “functions ideologically but … less hegemonically, for its program is not centralizing but eccentric: it creates space for interactions and diversity. In magical realist texts ontological disruption serves the purpose of political and cultural disruption: magic is often given a cultural corrective, requiring readers to scrutinize accepted realistic conventions of causality, materiality, motivation” (Zamora and Faris, 1995, p. 3). It is thus no accident that Jodorowski’s work, discussed previously, and *Papo & Yo* both have roots in the region.

It is this space for interaction and reflection on the notions of the individual self that poise magical realism as so apt for our purposes regarding symbolic enactment: it is often through a magical or supernatural act that ritual and myth are engaged upon or acted within. The “connections between magic and politics, between magic and self, between magic and action” (Hart and Ouyang, 2005, p. 8–11) are the design tools of this form of engagement. In the previous discussion of Jodorowski’s poetic acts, these can be read as a form of magical realism – the specific actions of the patient are afforded certain magical qualities as a phrase, action, or event. Within the act, these have magical qualities not normal in the real world, and this acts to set forward a consideration of the existential self. Later in this article, the player’s actions in *Papo & Yo* can be read in similar fashion, as certain magical abilities enable metaphorical action with symbolic meaning. This then forward the notion of using these types of enactments within games, with a lens on the capabilities and structures of magical realism. Or, as Bogost writes:

Likewise, magical realist authors like Gabriel García Marquez, Salman Rushdie, and Isabel Allende treat magic and myth as real, that is, they admit that the spectacular is real insofar as it actually comprises aspects of human culture. In cases like these, the philosopher’s tendency to abstract takes a backseat to the novelist’s tendency to specify. The result is something particular whose branches bristle into the canopy of the conceptual. Perhaps a similar strategy can both help illuminate the phenomenology of videogames and offer an approach to the pragmatic speculation on objects and their interrelations. (Bogost, 2008, p. 31)

### Symbolic Enactment in (Digital) Games – Existing Research

Games can afford indexical symbolic action to achieve transformative potential *via* presenting objects and facilitating actions that represent inner issues thus enabling players to act on those issues in a meaningful way. Often, though not always, these actions occur through a form of magic or ability that has outsized effects within the game world and thus invites reflection. But the research on this topic as a design methodology is thin. The most prominent work on games as the symbolic enactment of deeper, elusive themes is discussion of games of Murray (1997) as symbolic dramas. According to Murray (1997), the game structure becomes the tangible object onto which we can project abstract aspects of our lives and make sense of them.

In games, therefore, we have a chance to enact our most basic relationship with the world—our desire to prevail over adversity, to survive our inevitable defeats, to shape our environments, to master complexity, and to make our lives fit together like the pieces of a jigsaw puzzle. (…) Like the religious ceremonies of passage by which we mark birth, coming of age, marriage, and death, games are ritual actions allowing us to symbolically enact the patterns that give meaning to our lives. (p. 143)

Murray further refers to *Tetris* (Pajitnov and Pokhilko, 1984) as an example of a symbolic enactment: “Tetris allows us to symbolically experience agency over our lives. It is a kind of rain dance for the postmodern psyche, meant to allow us to enact control over things outside our power.” (Murray, 1997, p. 144).

Rusch (2009, 2016) explores Murray’s symbolic drama through the concept of “experiential metaphor”: the phenomenon of understanding a gameplay experience as a physical visualization of abstract ideas such as emotional processes or mental states. The concept of experiential metaphor is based on how – according to cognitive linguists Lakoff and Johnson (1980) – we understand and structure our experiences by way of experiential gestalts or image schemata. Engaging with a game on a mechanical, structural level can evoke association to similarly structured real-life experience, thus allowing players to explore intangible concepts in tangible, physical ways. This idea contains the psycho-magic of symbolic enactment in its essence. Rusch describes in detail how the grappling hook mechanic in *God of War II* (SCE Santa Monica Studio, 2007) acts as an embodied metaphor for transitions, and in particular compares it to dealing with a deteriorating relationship.

In *Making Deep Games*, Rusch (2016) notes that this mechanic spoke to her soul on a deep level, because it helped to make very concrete – visually, physically, emotionally – the different parts of the gestalt that she was being torn between (to stay and feel stuck, to jump and fail, to move on successfully). It also provided a much-needed sense of agency.

The progress on an emotional journey is often very hard to control or even assess. One day can feel fine while the next can feel emotionally catastrophic, and it is very unclear why this is so, how long it will last, and what to do about it. Being able to map these different emotional states onto tangible stages in the transition gestalt as represented in the game, and performing actions that would get her through them, helped Rusch feel calmer and more at peace, because it allowed an emotional (embodied and enacted) rehearsal of reaching the other side of her inner struggle. It may have been hard to measure, but she claims that this part of *God of War II* was transformational for her as its effects did transfer into real life.

Another work on symbolic enactment in games that deals explicitly with its transformative function stems from role playing game researchers focusing on live-action role playing games (LARP). Particularly relevant for a discussion of symbolic enactment is the work of RPG researcher and narrative designer Whitney Beatrix “Strix” Beltrán. Beltrán (2013, 2014) draws on depth psychology to explain how physically enacting various archetypal characters such as the trickster, virgin, mentor, or villain, can inform players’ understanding of themselves and contribute to personal development. She establishes strong parallels between ritual and LARP, declaring LARP a ritual space in which mythical themes are engaged in through direct participation and by way of acting out non-typical roles (Beltrán, 2013). This, Beltrán (2013, p. 93) explains, meets a need in our (Western) culture where “pervasive problems with identity and meaning have emerged.” She states further: “LARP is the West’s solution to addressing the need to explore and connect with other roles and states of physical and emotional being – essentially, to “live” myth” (Beltrán, 2013, p.95). She introduces the term “ego bleed” to describe how enacting archetypal character roles impacts the actual player’s psyche. By acting “as if,” the player becomes the character, experiences its archetypal energy firsthand, and takes some of this experience with them into real life, thus embedding it into their personality.

Ego bleed is a two way channel in which fragments of personality are passed between the player and their character. When a player enters into archetypal engagement during larp, it is therefore possible to experience ego bleed in which an archetypal characteristic inherent in a character type or role “rubs off” on a player. (2013, p. 96).

While there is relatively little existing research on these themes, this does not mean they are not relevant aspects of games’ transformative potential. A key difficulty is that their impact on players is highly dependent on a player’s receptiveness and the context of play (something the LARP and RPG community is very mindful of). While both games and ritual are capable of meaningful, transformative, symbolic enactment, one of the main differences between the two forms is what we expect of them and what meaning we ascribe to the actions we perform within them, as players may disregard the meaning of actions in games given the form of the media itself (Consalvo, 2007, p. 188).

### Caveat of Tapping Into the Transformative Potential of Symbolic Enactment in Digital Games

When asked by a skeptical interviewer how an indifferent mother could suddenly adopt the character of a loving mother in real life by performing a poetic act, Jodorowski (2010) points toward the crucial role of intent and expectation in the transformative process:

First of all, do not forget that my clients all suffered being dominated by their double. If they came to me, it was precisely because they felt bad in their role and sensed a completely different nature in themselves than the “original.” The process is founded, then, in a client’s real desire to change. (p. 50)

Kirmayer (1999, p. 453) similarly points-out in regards to the effectiveness of ritual in psychotherapy: “[t]he expectation of change has an effect that is separate from the specific content of cognitive reframing or the capacity of the prescribed ritual to promote change.” Research on the placebo effect also highlights the importance of the meaning that patients ascribe to the various aspects of the therapeutic process, including the doctor’s confidence, the color, the form (e.g., pill vs. vaccine), price point, side effects of the medication, etc. (Goodwyn, 2016). This surfaces an important caveat to the transformative potential of symbolic enactment in games. Playing a game from the perspective of enacting one’s most basic relationship with the world, as Murray (1997) proposes, could be a deeply moving, existential, transformative experience. But how often are players doing that? As stated above, for symbolic enactment to unfold its transformative potential, it requires the mind’s collaboration. If this collaboration is not facilitated through intentional design – through thematic/fictional/narrative choices or the game’s contextual framing – it solely depends on the player’s *a priori* receptiveness to the game, its characters, and/or particular experiential gestalts within it. While the grappling hook spoke to Rusch as an indexical symbol, this context does not exist to all players. To them, the grappling hook sequence remains an iconic symbol that exhausts its meaning in the representation of getting from one pillar to the next.

In general, we do not typically approach games with the same set of expectations as we might approach ritual, or as Jodorowski’s clients approached their poetic acts. In his DiGRA talk, Deterding (2016) argues compellingly that mechanics are not the (whole) message. The audio-visual and social framing of a game – e.g., as a serious, critical cultural artifact that belongs in a museum, such as Brenda Brathewaite’s (now Romero) *Train* (Romero, 2009), or as a game for children, such as *Playing History 2* (Serious Games Interactive, 2013) – shapes the expectations with which an audience approaches a game. This matters greatly for how the game is received and what message/experience it can effectively convey.

To harness symbolic enactment for transformation in games, designers must be mindful of how players facilitate meaning generation and how we are framing the gameplay experience as something that can potentially connect to a player’s real life. The Nordic LARP community has produced a useful concept in that regard: “bleed” (which is related but not identical to the “ego bleed” as noted earlier). Bleed describes moments where players’ “real life feelings, thoughts, relationships, and physical states spill over into their characters’ and vice versa” (Bowman, 2015). Bleed is a crucial factor in games’ transformative potential, but much like symbolic enactment, has remained underexplored in the context of digital games.

## The “How”: Conditions and Design Principles of Transformative Symbolic Enactment

If we thus assume that symbolic enactment can be effective, how can we design games for this purpose? What design factors can be identified that contribute to symbolic enactment’s transformative potential and can increase authenticity and inner balance? The following design principles and considerations have been derived from various theoretical and practical sources on transformative storytelling, ritual, poetic acts, and other experiential forms used in the context of counseling, psychotherapy, or personal development. They serve as the starting point for a design framework through which we can analyze existing games that contain indexical symbolic enactment in their gameplay and further help inspire new games that contribute to a meaningful life. This is intended as iterative theory building, which is then examined in the context of a case study as a means for generating foundational research: the design principles mentioned here are not complete. Furthermore, it is difficult to discuss symbolic enactment in isolation, since the context it is embedded in – the thematic and narrative framing – is important for it to unfold its transformative potential. Therefore, the following includes contextual considerations as well.

### Drawing on Three Types of Ritual to Inform Thematic and Narrative Framing of Indexical Symbolic Enactment

Referring back to the quote of Bugental (1990) in the introduction to this article, an authentic life is one in which, we are in harmony as fully as possible, including the conscious and unconscious mind. While external circumstances may not always allow for its full realization, we often know what “feels right.” A process of transformation in that sense leads from inner imbalance to balance, from confusion to clarity, from hesitation or tension to inner resolve, from grief or anger to peace, and from longing to fulfillment. Symbolic enactment is the process of acting through the conflict toward and including its resolution, but it is embedded into a bigger context of meaning and dramaturgical structure that further aids in bringing the transformation about. Rituals center around transition and, as such can be potent inspiration sources to facilitate such processes in game design – “changes that have happened, are happening, or may happen” (Beck and Metrick, 2012, p. 37). Parker and Horton (1996) extrapolated three main types of rituals from a phenomenological overview of rituals in various religious traditions, including Judeo-Christian, Asian, the Western Magical tradition and Shamanism: liberation rituals, transformation rituals, and celebration or commemoration rituals.

Liberation rituals use symbolic acts of removal or disengagement from obstacles to healing. Paradoxically, this can include destructive acts. When drawing on liberation as a theme for symbolic enactment in a game context, it is important to keep this theme at the forefront so that if destructive acts are used, players stay aware of their purpose – that is, being a vehicle for healing – and those acts do not devolve into self-serving, gratuitous violence. Liberation rituals can be encapsulated in individual moments of a larger game (possibly with a different overall theme).

Transformation rituals, which commonly describe rites of passage, are naturally processual.

Through them [i.e. transformation rituals], something new is birthed, affirmed, blessed, and empowered. In transformation rituals, the elements of initiation and blessing are coupled. These two elements go together naturally and necessarily, as do birth and nurturance. (Parker and Horton, 1996, para. 29)

In their discussion of ritual, Beck and Metrick (2012, p. 37) only focus on transformation rituals and further distinguish them into rituals of “beginnings, mergings, cycles, endings, and healings.”

Celebration and commemoration rituals are associated with religious worship, anniversaries, birthdays, and local cultural holidays. The term worship is particularly relevant here and should be understood in its original sense of appreciating the “worth-ship,” or worthiness, of something. “In commemoration rituals something valuable is preserved or honored through remembrance or celebration” (Parker and Horton, 1996, para. 36). Rusch (2018) provides examples for how these types of rituals can inform game mechanics.

### Embedding Symbolic Enactment Into Metaphorical Context

It has already been stated that the language of the unconscious consists of symbols and imagery. Mental images are essential for the placebo response and play an important role in narrativizing inner conflicts. Metaphorical stories promote meaning generation, and finding meaning in a psychological or physiological issue greatly contributes to the ability to overcome it. Images tend to be loaded with emotion, which impacts their transformational potential; we cannot be changed by something we do not care about (Goodwyn, 2016). Imagery in existential, transformational game design is thus not only important for the symbolic action itself, but also for the design of the context into which it is embedded. Rusch highlights the use of myth and mythical themes in game design and notes how we can explore the unconscious for mythical themes through dream work and active imagination (Rusch, 2018, 2020). Myths are metaphorical stories and they often accompany or frame ritual enactments.

Campbell (2004, p. xix) states: “[r]itual is simply myth enacted; by participating in a rite, you are participating directly in the myth.” When ritual is used in psychotherapy, the myth that is being enacted stems from the client’s story (and in humanistic, existential psychotherapy and Jungian approaches, the client’s story is investigated in regards to its more universal/transpersonal mythical themes). This is also true for Jodorowski’s poetic acts; they are not just symbolic acts, they are enactments of transformational stories in which the client is transformed into someone else, a “truer” version of themselves. Although, since the client “knows” their story, the individual context is assumed. When it comes to designing opportunities for symbolic enactment for an audience of anonymous strangers – typical of game design – it is important to thematically/narratively contextualize the symbolic action in a way that supports transformation. A metaphorical approach points toward a deeper meaning beyond the game’s surface (Rusch, 2016) and, depending on how obvious the metaphorical nature of the game is, can orient players toward the symbolic nature of the action as well. Metaphors are also ideal stylistic devices to capture intangible concepts, such as emotions or inner conflicts, because they make abstract ideas concrete (Lakoff and Johnson, 1980; Johnson, 1987).

### Promoting “Change” Through Contextual Mechanisms

Readiness and processing are crucial for change. Ritual and other experiential forms that target personal transformation have identified mechanisms to promote both. Pitkänen (2019) compares four types of experiential “forms of change” – psychodrama, sociodrama, playback theater, and educational LARP – and identifies that the structure of “warm up,” “drama” and “processing” are common to them all. While the “drama” part differs considerably across the four forms, warm up (which is used to reach a spontaneous, playful state that allows the exploration of new ways of acting and being) and processing are similar.

This structure corresponds to ritual performance which also includes entering a sacred space – a space “set apart” in some way from daily life – and performing the ritual and then deepening the experience through some kind of grounding action that concludes the experience (Beck and Metrick, 2012). Nordic LARP, known for tackling difficult themes and its transformative potential, employs similar mechanisms by commonly preparing players through an introductory workshop. A debriefing of the gameplay experience occurs through another workshop at the end of the ritual performance that aids in integrating what players have learnt about themselves and the topic during play and allows them to “de-role” and get back to daily life afterward (Bowman, 2014).

What does that mean for digital games, where the situation of the player is entirely different? Unless the game is played in a facilitated context, like in a therapist’s office, school, hospital, etc., the player is left to their own devices with respect to easing into and out of the game’s magic circle. Individually, they must emotionally process and integrate their game experience into other aspects of their lives. Since designers have little control over the contexts within which their games are played, the best tool to facilitate intention setting is likely narrative, either explicitly or through visual/auditory means.

### Psychological Resonance in the Design of Symbolic Enactment

Rusch (2020) has discussed at length the concept of psychological resonance with regards to identifying mythical themes for game design. This has direct relationships for indexical symbolic enactment. She notes,

Based on psychiatrist Erik Goodwyn’s (2012, 2016, 2018) highly interdisciplinary research, psychological resonance refers to a deep, unconscious recognition and activation of archetypal patterns through symbols and imagery. Psychological resonance is at the root of the same kinds of mythic, symbolic and ritual ideas popping up time and time again all over the world, across all cultures. It is the key to understanding “what makes one ritual more likely than another to be repeated across generations?” (Goodwyn, 2016, p. 33). It’s about *what* speaks to us (as humans) and *why*, on a deep, unconscious and universal level. Understanding and harnessing psychological resonance is key to designing transformative existential games, because of their emphasis on awakening our authentic self, aligning us with what rings true for us, so we can identify our own pathways to bliss, uncoerced and to our own terms. (Rusch, 2020, p. 9)

Goodwyn (2016), drawing extensively on folklore studies, lists the following criteria for psychological resonance:

I propose that the most resonant expressions are likely to have some or all of the following:

1. Minimal counter-intuitiveness (Barrett, 2007), meaning that they have only a few unusual or strange elements and so stand out, rather than have too many or too few counter-intuitive elements. Examples: talking animals, flying carpets, dragons (…)
2. Emotional evocativeness (Panskepp, 1998). Examples: stories involving basic human attachments or evoking basic emotional responses such as fear, anger, lust, and so on.
3. Sensual vividness, with a tendency toward extremes. Examples: castles of gold, mountains of crystal, brilliant lights, absolute darkness, and so on.
4. Indeterminacy of time and space. Examples: “long ago in a far-away land” – evocative of an oceanic feeling.
5. Biasing toward middle-level categories. Examples: “sword” rather than “weapon” (too abstract) or “quillioned pattern-weld blade with Brighthampton scabbard and cross” (overdetailed)
6. Low complexity of characters and motivations. Examples: the most beautiful in the land, the king, animal gods, the thief, and the beggar.
7. Rhythmic and prosodic/musical elements. Examples: “magic mirror in the wall.”
8. Simple plots with reversals and/or irony. Examples: nothing is as it seems, plot twists, the slow animal beats the fast animal, and so on.
9. Apparent interconnection of events. Examples: things always occurring “just in the knick of time”, and so on.

Non-resonant expressions will be: overly counter-intuitive or overly mundane, emotionally detached or frustrating, sensually vague or abstract, specific in time and space, contain over-specific or over-general categories, be internally complex or ambiguous, will lack any rhythmic or poetic qualities, will lack a clear plot (…). (pp. 37-38)

In regards to indexical symbolic enactment specifically, emotional evocativeness, sensual vividness, minimal counter-intuitiveness, and low complexity of actions and objects involved in symbolic enactment seem particularly important, as the other criteria are more directed toward narrative expressions. If actions are too complex, convoluted, elaborate, hard to understand, and involve intricate objects, props, tools, or weapons whose functionality is unclear or complex, it is hard to relate to them intuitively. Plants, seeds, graves, family pictures, fire, water, earth, snakes, etc. are symbols frequently used in ritual and Jodorowski’s poetic acts because of their intuitive, sensual, emotional qualities that are further tightly coupled to the theme that is being enacted.

## Indexical Symbolic Enactment in Action: A Case Study of *Papo & Yo*

The following section analyzes *Papo & Yo* (2013) in regards to how it employs and contextualizes indexical symbolic enactment that can increase authenticity and inner balance. We employ auto-ethnography as a method for this analysis and as such it is subjective. The basis for our discussion of the game’s most symbolically potent gameplay and transformative aspects is our personal experience of it, both as players and as designers.

There are many game mechanics in this game that can be described as indexical symbolic enactments. Only a few of them, however, or only specific parts of the game, truly feel like they promote inner change by giving the player a handle on an inner conflict. What is it about these mechanics/parts of gameplay that stand apart from the rest and take on this special quality that we can also find in ritual or Jodorowski’s poetic acts? How do these specific sections of the game provide an authentic sense of agency around an intangible issue that ignites an inner shift toward greater harmony?

### Enacting “Liberation”

*Papo & Yo* is a puzzle platformer adventure game, based on Minority Media’s founder and the game’s lead designer Vander Caballero’s childhood. He created the game as an expression of how he overcame his personal trauma of living with an alcoholic father and as a way to help others do the same. “Games can really help people” Caballero said in an interview (Donnelly, 2017), “they can help people to heal.” While designing with such intent in mind is not necessary for games to have such impact on players, it is one reason why this game was selected as a case study. The game affords an experience of *liberation*. Its overall narrative structure is akin to a liberation ritual – enacting an emotional journey from inner conflict to resolution, from feeling “cursed” and responsible to help someone who does not want to be helped to “being free.” This journey further culminates in a final act that is explicitly ritual-like.

### Metaphorical Approach

*Papo & Yo* is a (mostly) metaphorical representation of what life is like with an alcoholic father. The player takes the role of a young boy, Quico, who lives in a Brazilian favela with Monster. (Monster, here, is used like a name). It begins as a cutscene with a literal depiction of Quico’s home, where Quico hides in the closet from another one of his father’s drunken rages. As the boy cowers in the dark, a white spiral portal drawn in magical chalk appears on one of the closet walls. As Quico – now under the player’s control – steps through the portal, the game transitions to a metaphorical space that can be interpreted as Quico’s inner world, which looks like his real environment but enriched with magical/fantastical elements and is characterized by imagination and creativity. In this space, Quico gains freedom of movement through manipulating the environment by way of chalk drawings, which are created by a girl whose relationship to Quico remains unclear, but might be a sister. The gears, levers, springs, and lines she draws allow for surprising, delightful and whimsical ways to reshape and restructure the world. Caballero reports about his own childhood: “I started being creative when I was a kid, and I coped with the difficult things I was going through with creativity” (Donnelly, 2017). The puzzle platformer gameplay can thus be understood as a metaphor for using imagination as a way to navigate difficult emotional terrain.

The important function of creativity is further made tangible in the gameplay by Quico’s toy robot Lula, who protects Quico by catching his falls and who “lends him wings.” With Lula on his back like a jet pack, Quico can jump higher and glide short distances. Lula can also be sent off alone as an extension of Quico, operating gears, levers, and buttons that are otherwise out of reach.

However, imagination and creativity are just ways of coping. They cannot eliminate Monster and its influence on Quico’s inner world. Early on in the game, the girl yells at Quico that he is “cursed.” We then learn through two cut scenes set in the physical world that Quico carries guilt for a drunk driving accident he witnessed, in which his father killed someone. The girl assures Quico that the accident wasn’t his fault. That killing is Monster’s burden. She then informsQuico that he can cure Monster by bringing it to a Shaman. This establishes the game’s goal and its conflict: steering Monster through the favelas, thus creating a path for it, while keeping it away from frogs. Hen Monster consumes frogs, it turns into a flaming beast of destruction and attacks Quico. Monster can be lured with some orange fruit that is found all over the environment. When it eats enough of it, it falls asleep. When it falls asleep on the Centipede – a slat of wood with many scrawny, magical chalk legs – the Centipede will carry Monster to where Quico needs it to go. Monster is mostly passive. It can even be (passively) helpful. Its big belly can act as a jumping board for Quico to launch him to higher up areas. At one point in the game, Quico can kick a ball toward Monster and Monster kicks it back. It remains the only moment of tender interaction between the two and as such feels highly meaningful – a reminder of what the relationship *could* be like, if things were different.

With this metaphorical set-up, *Papo & Yo*’s gameplay can be seen as indexical symbolic enactment of a conflicting, emotional journey through feelings of guilt, responsibility, fear, and the longing for a real relationship with the father, contrasted by the experience of agency and joy enabled by creativity. The inner conflict intensifies as the journey goes on and Monster, in a fiery rage, first destroys Lula and then kills the girl. Both are strong, symbolic acts of how the father’s addiction impacts the well-being of Quico’s and those around him, his ability to feel safe, empowered, and joyful, and this impact becomes experientially tangible to the player by taking away the mechanics that are afforded by Lula.

The game’s moment-to-moment gameplay is an enactment of the conflict itself. The majority of the game does not promote authenticity and inner balance. It is an emotional and experiential build up toward the final scene, in which Quico/the player enacts liberation from Monster. Once on top of the mountain where the shaman is supposed to be, a disembodied voice – his inner wisdom? – tells Quico that he has done well, but that there is no shaman. There are only Quico’s forgotten memories; the memories of all the horrible things Monster did. These memories take the form of stone statues that, when Quico pulls magical chalk levers attached to them, transform into literal depictions of what they are meant to represent. From them, Quico is reminded of how he played with Lula in real life; of father’s drinking; of how her father attacked the girl (that she exists in the literal world is a clue that she might be someone from his real life); and of how her father beat Quico.

Pulling the lever and transforming the metaphors into their literal sources can be interpreted as a process of confronting the reality of living with Monster – of seeing things as they are. While the player is enacting this part and the enactment is based on indexical symbolism, its function is mainly to communicate to the player what is happening inside Quico, rather than giving the player first hand access to his inner processing. It is a snapshot of what is happening within Quico, but the action itself – pulling the levers – carries little emotional weight. It does not feel like the player is given agency over an inner process the way ritual does, because lever-pulling does not provide agency over the elusive and intangible; it is merely functional. In other words, this mechanic is simply a way to force interactivity.

With this acknowledgment of reality freshly in mind, Quico is informed by the disembodied voice that there is no cure and that Quico needs to let go of Monster. This initiates the last part of the game that feels very much like a liberation ritual. A magical chalk line appears and leads straight to a huge rock. The rock crumbles, possibly symbolizing an inner breakthrough. Quico enters a surreal space – the magic circle of the ritual – that only features the Monster’s house floating in the air and some other disjointed platforms on which powerful symbolic actions that represent letting go of Monster are about to take place. There is no more puzzle platforming. The game also becomes slightly more literal in that it depicts alcohol through bottles, and not just the frogs, revealing the metaphor.

The first platform features two huge tubes. When activating a lever, bottles pop out of one tube. Quico/the player then carries the bottles to the other tube that sends them to the platform where Monster is. The bottles appear there as frogs. Monster consumes the frogs and becomes the flaming beast, setting things around it on fire. This action of taking a bottle, putting it into a tube, and feeding it to Monster has to be repeated several times before the bridge that connects one platform with the next is completed and Quico/the player can move on to the next platform and the next step in the process of letting go.

Delivering the bottles is a powerful symbolic enactment of giving responsibility for destructive behavior back to Monster. As the player pushed bottle after bottle through the tube and saw Monster eagerly consume the frogs – destroying itself and everything around it through the flames – Rusch noted her inner voice saying things like “There! You want it? Take it! All of it! Do as you please! I am done trying to help you.” While Rusch has not lived with a substance abuser, she has been in a co-dependent relationship before and tried very hard to help someone to the point of losing a part of herself. While understanding the metaphor of the bottles intellectually, the real power of enacting this part of *Papo & Yo* came from mapping the experiential gestalt (Lakoff and Johnson, 1980) of the metaphor and its symbols onto this former relationship. The specific symbols used, in this case “bottles” as “alcohol,” are what Jung would call “signs,” because on this interpretation level, they refer to something known: alcoholism. But within the structure and context of the whole act, these symbols can point toward a wider range of elusive concepts. Even the reading of “bottle” as “alcohol” is too limiting in the context of the whole act and points toward the more encompassing and fuzzier concept of “choices/responsibility for self and others.” As such, the bottles become indices for any burden one is carrying unjustly and to one’s own detriment. They tap into a deeper level of experience that is more universal (and thus more broadly relatable) than the specific story Vander Caballero chose to tell in *Papo & Yo*. This allows the game to take on a mythical component, becoming a road map for the human experience that carries wisdom beyond the particular narrative.

The next step in the liberation process is mechanically similar to the first one; there are again two large tubes that connect the platform Quico/the player is on with that of Monster. Instead of bottles, there is a heap of rag dolls that look like the girl Monster killed in one of its rages. Quico/the player picks up the doll, throws it in one of the tubes and can then direct where it lands on Monster’s platform. On Monster’s platform, the doll comes to life and tries to run away but is grabbed by Monster and eaten. Every time one “sacrifices” a doll in this manner, the environment on Monster’s platform changes and a bridge to a lower platform gradually manifests. Once the bridge is complete, one has to drop the next doll onto the elevated platform. The doll, now alive, will run away and be chased by Monster onto the next and final platform.

The rag doll scene feels surreal and both authors had a hard time connecting to it emotionally. Delivering the doll made us feel guilty and uncomfortable rather than empowered. The specific nature of this enacted sacrifice – to fulfill the gameplay purpose of luring Monster to the final platform – is incongruent with the overall transformative process of letting go of Monster. There are too many symbolic elements that are not intuitive. Intellectually, the player must relive one of Monster’s worst acts in order to work up the resolve to finally let Monster go, but this interpretation exists only on a cognitive level. This cerebral focus, and the absence of strong emotion while playing this scene, is an indication that the symbolic enactment of this scene is not successfully speaking to the unconscious. It is an intellectual exercise, not felt on a deeper level and thus arguably lacks much transformative power.

The game’s ending, however, is very powerful. Once Monster arrives on the final platform, it falls asleep on the Centipede that is already waiting there for it. To complete the journey, Quico/the player pushes the Centipede toward the edge of the platform to an endless sea of clouds down below. This simple act of pushing Monster over the edge and seeing it disappear is deeply and psychologically resonant. It moved both authors to a cleansing, meaningful sadness. The actual story of the game receded into the background, and the act of pushing Monster away became a much more encompassing, indexical symbol for all complicated good-byes and torn relationships. For Rusch, in particular this resulted in a reflection and sense of peace; something inside her had shifted and allowed a realization to sink in, or at least anchor it more deeply, that she did not need to be other peoples’ savior.

### How Does *Papo & Yo* Promote “Change” Through Contextual Mechanisms?

The authors played the game at home and had only passing knowledge of the subject matter, so there was no external context set up to facilitate change. This is a very different situation than a client who voluntarily enters any kind of psychotherapy. We had no particular expectation to be transformed by this game, and the fact it occurred regardless is owed largely to the game’s narrative. As players, we could be gradually introduced to Quico’s struggles and align with his inner conflict, therefore symbolically enacting its resolution with him. After the rollercoaster of emotions previously described, each of the authors sought to process what had occurred. For Rusch, she felt a bit lost and alone and was lucky to be able to process and debrief the experience with her husband later that day. For Phelps, he engaged in an online discussion and debrief with others who had also played the game. How can digital games facilitate processing? The tiniest nod toward a transfer to real life is the game’s literal framing; it starts in the physical or “real” world and Quico/the player returns to it once the journey is over. This can help a player make the connection to their own life to contemplate how what they just experienced in a fantastical setting applies to their daily, real life struggles.

### Psychological Resonance of Symbolic Enactment in *Papo & Yo*

When Goodwyn (2012, 2016) discusses psychological resonance, he establishes it as an empirical concept. We only know whether an expression is truly psychologically resonant if it has stuck around for a very long time and survived the process of cultural transmission. Rusch (2020) has argued that we can use the criteria, listed above in this article that characterizes psychologically resonant symbols, imagery, and narratives as guidelines to create new mythical games. By designing games that harness the strategies (symbols, imagery, etc.) that have been empirically proven to be psychologically resonant through their longevity in folklore studies and mythology, we can aspire to create psychologically resonant games. It is highly impractical to conduct longitudinal playtesting in the scale that would be necessary to empirically assess whether a game truly *is* psychologically resonant in the ambitious way Goodwyn understands the concept. We note instead that aspiration to evoke psychological resonance is the extent of what we can currently achieve as an alternative to empiricism, given the fact these approaches are just emerging more formally in the design literature. Thus we employ an analytical lens to examine *Papo & Yo’s* symbolic enactment, with *indications* of resonance. We then argue for the presence of such resonance given our responses during this playtest and subsequent reflection.

#### Moment-to-Moment Gameplay

*Papo & Yo*’s moment-to-moment gameplay consists of two, salient indexical symbolic enactments: “puzzle platforming” as an enactment of navigating through an inner landscape by way of imagination and creativity; and “frog elimination” – getting to the frogs before Monster does and preventing consumption. Both of these mechanics make thematic sense within the game’s metaphor and help to reinforce what the game is about experientially; they align the player with their avatar, Quico, and make Quico’s inner conflict tangible on a moment-to-moment basis. They thus belong to the “warm up” phase of the transformational process, orienting the player toward “what’s wrong” in the game world rather than to a phase of conflict resolution. For this warm up phase to be impactful, though, it must emotionally engage the player.

The factors for psychological resonance that seem particularly important in regards to symbolic enactment are emotional evocativeness, sensual vividness, minimal counter-intuitiveness, and low complexity of the actions and objects involved (Goodwyn, 2016). Puzzle platforming and frog elimination fulfill this criteria to various degrees through gameplay, artistic representation, and sound design. While physical modes of symbolic enactments such as ritual, poetic acts, and LARPs have many advantages over digital games in regards to promoting transformation, affording sensually vivid experiences is one of the great strengths of digital games. Metaphors and symbolic elements can be lavishly represented through both representation and interaction. However, careful thought is required in combining mechanics with representation; while the puzzle component is not overly difficult in *Papo & Yo*, it still requires some mental acrobatics which detract from psychological resonance by shifting the focus of the experience from the emotional to the cognitive realm.

The frog elimination sequences are less complex – all the player has to do is run up to a frog, grab it, and smash it against a wall – but the image of using green, harmless-looking frogs to represent alcohol is not intuitive and therefore not immediately resonant. The only reason for a player to dread the frogs is because of the rule system that determines their function in the game: if Monster eats them, there is hell to pay. Finding a symbol that conveys the dangerous, destructive aspect of alcohol more strongly would have enhanced the overall psychological resonance of this scene. Then again, the gameplay itself, through the hunt for the hopping creatures and the grabbing and smashing of them, does not share salient, experiential parallels with the much more complex dance around an alcoholic. This can include the tangible component of finding and emptying hidden bottles but also has the emotional component of being afraid of the alcoholic’s moods and the need for caution in their presence. Frog elimination is a very “gamey” way of representing this aspect of life with an alcoholic. It focuses on the physical challenge rather than addressing the psychological complexities. By “gamey” we mean it foregrounds the entertaining challenge involved in performing the action (i.e., luring and trapping Monster without being attacked) and backgrounds its meaning. This is not inherently “wrong” or “bad” – it lies in the nature of game design to abstract complex systems to a degree where they become playable (Juul, 2007) and to focus on giving players agency. It is hard to imagine a game in which every action carries a lot of symbolic weight and to sustain an awareness of this symbolism across several hours of gameplay. There are, however, specific scenes in *Papo & Yo* that stand out in this manner from the moment-to-moment gameplay, and it is those scenes that have the highest transformative potential.

#### Ritual-Like Enactments

There are four scenes in *Papo & Yo* that stand apart from and disrupt the flow of the regular gameplay. They slow the action toward the game’s goal at particularly meaningful points in Quico’s emotional journey and are structured in distinct steps. This gives the moments a ritual-like quality and makes them deserving of special attention as they carry the bulk of the game’s transformative potential in regards to indexical symbolic enactment. Such examples include reviving Lula, and the three stations of the “letting go” sequence at the end of the game: “bottles,” “rag dolls,” and the actual “letting go.”

##### Lula’s Revival

In examining the three types of rituals discussed above, reviving Lula shares salient characteristics with a transformation ritual, from death to life. Lula is placed on an altar. Then, Quico/the player has to lure Monster to a trap; the trap engulfs Monster and “squeezes the anger out of it.” This anger – symbolized by a single, orange flame – makes its way to Lula on the altar and infuses the broken robot with life. This process has to be repeated three times, with each flame that is squeezed from Monster being absorbed by Lula. The theme of bringing an important companion back from the dead is obviously emotionally evocative, and the player is thus empowered. The ritual allows Quico/the player to reclaim not only their companion, but everything it stood for: freedom of movement, agency, and joy.

What challenges the ritualistic quality of the scene and undermines its symbolism, though, is the process of eliciting the life-giving flame as well as the imagery of the flame itself; the mechanic has a decidedly “gamey” feel to it. The emphasis is not on performing an action that symbolically engages an inner process in a way the unconscious can intuitively understand but is rather merely an interesting solution to the design problem of making the Lula revival scene playable in a way that connects gameplay to story. While the imagery is vivid, the sequence of actions performed is too complex and requires too much intellectual interpretation to resonate on a deeper, emotional level. Further, the flame that is elicited has the wrong color. When Lula is active, it produces a little purple spark. The orange colored flame is connected with Monster in a fiery rage. Why should an orange flame with its destructive associations bring life back to Lula? The power of ritual is based on analogical connections between its symbolic elements and what they stand for. This is instrumental for governing expectations around the “magical” functioning of symbols in ritual. Using the orange colored Monster flame to restore life to Lula suggests that Lula is now connected to Monster in some essential (and troublesome) way, which is not the case.

The design is guided by the question “what can the player do that also ties back into the narrative?” rather than by “what action is a symbolic expression of an inner process that would have the strongest impact?” It is possible to emphasize the latter over the former; when Quico has to face all the bad things Monster has done to accept that Monster is beyond help, this abstract process is represented by way of stone statues that stand for memories. Something similar could have worked for the revival of Lula: Quico could have recovered life force flames from good memories of playing with Lula, while possibly having to avoid Monster. This would have allowed for similar gameplay, but with less distracting detail and more metaphorically fitting imagery. The concept of “restoring life” can be more intuitively connected to harvesting a positive essence from positive memories rather than to squeezing out anger from an abuser. This is just one possible alternative.

##### Letting-Go Sequence

The final part of the game is the “letting-go” sequence, which consists of feeding bottles to Monster, sacrificing the rag doll and pushing Monster into the abyss. Feeding Monster in this way and seeing Monster burst into flames, consequently destroying everything around it, is an emotionally evocative scene with sensual vividness. Actions and imagery are congruent with the inner process of handing back responsibility and coming to grips with the fact that the other person is truly unwilling to change. Feeding bottles to Monster has low enough complexity to be intuitively understandable as the symbolic act of handing responsibility over. It is as simple as “there, you have it.” Thus, this step of the letting-go sequence as a whole has high potential for psychological resonance.

The rag doll scene lacks this intuitiveness of meaning: it is pragmatic from a gameplay perspective but not psychologically congruent. The function of the rag doll scene in the greater structure of the “letting go” sequence is to lure Monster to the final platform. It feels like using the rag doll was an attempt to tie in established, meaningful symbols into the gameplay to keep the game’s theme and narrative in the player’s mind, but without deeper consideration.

Finally, Quico/the player lets go of Monster by pushing it into the abyss. This is the shortest of the ritual-like scenes in *Papo & Yo*, but the most powerful. It focuses on letting go, which is not compromised by any design ambitions to make this more interesting with regard to gameplay. The connection between pushing Monster over the edge and saying goodbye is straightforward, intuitive, and emotionally evocative. The imagery of sleeping Monster, arms folded over its chest as if dead, completely resigned to its fate, is intuitive and cathartic. Enacting this scene sends a psychologically powerful resonating message to the unconscious, supported by the audio-visual design: “Farewell, Monster. You’re no longer my problem.” The struggle between hope, longing for a loving relationship with father, and feelings of guilt and responsibility has been resolved through this act. The game reaches a sense of inner peace that is transcendent with its players and their life experiences.

## Conclusion

Drawing on interdisciplinary research, and inspired by ritual use in psychotherapy and Jodorowski’s poetic acts, we argue that indexical symbolic enactments of inner conflicts in games and digital media can send powerful signals to the unconscious mind, thus contributing to greater authenticity and inner balance. Designers can directly facilitate indexical symbolic enactment for existential and transformative gameplay. This article proposed a theoretical framework including design guidelines meant to inspire designers who want to create games that contribute to a meaningful life. Those guidelines can be summarized as (1) identifying a narrative theme and structure that is inspired by the three types of rituals: liberation, transformation and commemoration/celebration; (2) embedding symbolic enactment into a metaphorical environment; (3) considering contextual mechanisms that allow players to “warm up” to the inner conflict modeled in the game and promote its “processing;” and (4) designing for psychological resonance by aiming for emotional evocativeness, sensual vividness, minimal counter-intuitiveness, and low complexity of actions and objects involved in the enactment as noted by Goodwyn (2016).

To illustrate this concept, the authors examine *Papo & Yo* through the lens of this design framework. We examined moment-to-moment gameplay, as well as four special moments in the game that stand out from the rest due to their ritualistic quality. The analysis showed that there is significant potential for indexical symbolic enactment, but that design decisions did not always favor symbolic congruency and psychological resonance for the greatest transformative impact. *Papo & Yo*, while in many ways playing with spiritual motifs, and being both inspired by personal transformation as well as intending to inspire it in others, has been conceived primarily from a “game design” perspective. This is neither wrong nor bad, per se, but means that less emphasis has been placed on the design of opportunities to strengthen the intuitiveness and psychological resonance of symbolic actions. Alternatively, an existential, transformative game design framework aims to facilitate both the design of games that are both engaging but also aware of the transformative potential through indexical symbolic enactment. This work thus hopes to inspire game designers to experiment with this approach and thereby lead to the creation of projects that contribute to a meaningful life for players by fostering authenticity and inner balance.

## Data Availability Statement

All datasets presented in this study are included in the article/supplementary material.

## Author Contributions

The authors have been working collaboratively toward the bigger framework of existential, transformative game design for many months. DR wrote the original draft of this pillar of the larger theoretical design framework, while AP helped ground it in the background research and contributed throughout to framework design and collaboration. Both authors contributed to the article and approved the submitted version.

### Conflict of Interest

The authors declare that the research was conducted in the absence of any commercial or financial relationships that could be construed as a potential conflict of interest.
